# Developing the protocol for the evaluation of the health foundation's 'engaging with quality initiative' – an emergent approach

**DOI:** 10.1186/1748-5908-3-46

**Published:** 2008-10-30

**Authors:** Bryony Soper, Martin Buxton, Stephen Hanney, Wija Oortwijn, Amanda Scoggins, Nick Steel, Tom Ling

**Affiliations:** 1 Health Economics Research Group, Brunel University, Uxbridge, Middlesex, UB8 3PH, UK; 2 ECORYS NL, P.O. Box 4175, 3000 AD Rotterdam, the Netherlands; 3 RAND Europe, Westbrook Centre, Milton Road, Cambridge, CB4 1YG, UK; 4 School of Medicine, Health Policy and Practice, University of East Anglia, Norwich, NR4 7TJ, UK; 5 NHS Norfolk, Lakeside 400, Thorpe St Andrew, Norwich, NR7 0WG, UK

## Abstract

In 2004 a UK charity, The Health Foundation, established the 'Engaging with Quality Initiative' to explore and evaluate the benefits of engaging clinicians in quality improvement in healthcare. Eight projects run by professional bodies or specialist societies were commissioned in various areas of acute care. A developmental approach to the initiative was adopted, accompanied by a two level evaluation: eight project self-evaluations and a related external evaluation. This paper describes how the protocol for the external evaluation was developed. The challenges faced included large variation between and within the projects (in approach, scope and context, and in understanding of quality improvement), the need to support the project teams in their self-evaluations while retaining a necessary objectivity, and the difficulty of evaluating the moving target created by the developmental approach adopted in the initiative. An initial period to develop the evaluation protocol proved invaluable in helping us to explore these issues.

## Background

The quality of healthcare and the role of professionals in leading improvement vary substantially [1-4]. In recent years many countries have initiated large-scale quality programmes, and there has been a wide range of quality improvement initiatives and wide variation in terms of their impact and success [5]. In the UK, the thrust of change established in the National Health Service (NHS) Plan in 2000 [6], and reiterated in 2004 [7], is now being continued through the Darzi Report, which aims to put quality at the heart of the NHS, empowering staff and giving patients choice [8]. This developing policy framework has been accompanied by a continuing debate about how quality improvement should be conducted and evaluated [9,10], a debate that has focused not only on the methodologies to be adopted but also on the need to work within appropriate theoretical frameworks, such as organisational or behavioural theory.

One influential review of the literature on the effectiveness of different activities intended to improve clinical quality (such as guideline dissemination and implementation strategies) was undertaken by Grimshaw and colleagues in 2004 [11,12]. The quality of many of the studies identified was poor, and the review acknowledged many unknowns, but it was clear about the potential benefits to be gained from engaging clinicians in quality improvement and about the difficulties in delivering and evaluating this. Using the methods proposed by the Cochrane Effective Practice and Organisation of Care Group, this review worked within the standard approach for evaluating medical interventions, *i.e. *that the best way to get to the 'truth' about effective care is via a randomised controlled trial (RCT).

But there is another side to this coin. While acknowledging the merits and achievements, of the RCT, its limitations for evaluating complex social changes such as health care quality improvement initiatives have been recognised for some time [13,14]. Before a quality improvement initiative can be generalised to other settings, we need to know why the initiative works, as well as whether it works. The debate is about epistemology, about what sort of evidence should be sought, underpinned by the argument that there should be a strong relationship between what is studied and how it is studied. And in the context of quality improvement Berwick talks about pragmatic science, by which he means methods of observation and reflection that are systematic, theoretically grounded, often quantitative, and powerful, but are not RCTs [15]. But if RCTs are not the best approach, what is? As a worked example of an alternative approach, this paper discusses the development of the protocol for evaluating a complex, multi-component, multi-site, quality improvement initiative.

### The 'Engaging with Quality Initiative'

In 2004, The Health Foundation (an independent UK charity working to improve the quality of healthcare across the UK and beyond) invited national professional bodies and specialist societies in the UK to bid for funds for projects to engage clinicians in making measurable and sustainable improvements in the quality of clinical care under the Engaging with Quality Initiative (EwQI). The three objectives of the EwQI are given in Table 1, and the criteria for the selection of the projects in Table 2.

**Table 1:** EwQI objectives

• To engage clinicians in leading quality improvement projects that will achieve measurable improvement in clinical quality
• To identify effective strategies for clinical quality improvement that can be replicated and spread across the healthcare systems

• To increase capacity for clinical quality measurement and improvement in the UK by developing the infrastructure and skills within professional bodies

**Table 2:** Selection criteria for EwQI projects

• clarity of aims and objectives
• scope for achieving significant improvements in clinical quality in the proposed area of care

• attention to patients' definitions and experience of quality of careg

• arrangements to secure clinical buy-in and national coverage

• quality of the technical aspects of the project including sampling, standards of data definition and verification, arrangements for clinical interpretation of findings and clinical feedback in reports, access to methodological and analytical expertise

• proposed arrangements for ownership and disclosure of data and results

• strength of proposed strategies for quality improvement interventions and their evaluation

• strength of proposed evaluation plan for quality improvement interventions (all applications) and measurement and reporting system (full cycle projects only)

• plans for communicating findings to the wider healthcare community and the public

• robustness of proposals to secure sustainability and spread

• capacity of the applicants to deliver completed projects within budget.

The immediate inspiration for the EwQI came from Leatherman and Sutherland's book 'The Quest for Quality in the NHS: A Mid term Evaluation of the Ten Year Quality Agenda' [4]. This concluded that clinicians in the UK are attentive to the need to improve quality, but are not fully engaged. The Health Foundation's decision to invest in projects run by professional bodies or specialist societies reflected Leatherman and Sutherland's findings that clinicians listen and learn best from their peers, and that these bodies have a legitimacy and authority that commands clinicians' respect. This decision recognised a potential role for these bodies notwithstanding Leatherman and Sutherland's reservations about the role they have played in the past and about whether they all possess the skills and capacities to play a leading role in engaging professionals in quality. Other considerations that shaped the EwQI were: the need to base clinical improvement on sound evidence about best practice and to build, where possible, on existing high quality audits or other performance measurement and reporting systems; the need to involve users (patients and carers) from start to finish; and the importance of developing sustainable improvements in quality.

The Health Foundation's general thinking about how to improve quality in healthcare also influenced the EwQI in two other ways: a developmental approach was adopted, and an evaluation was planned. The latter was to be evaluation at two levels – an external evaluation of the EwQI as a whole, and a set of self-evaluations at project level. The rationale throughout was the same: the Health Foundation wanted to encourage all those engaged in the initiative (including themselves) to learn and adapt as they went along.

Three teams were commissioned to support the project teams during the initiative: an EwQI support team, whose brief was to help the project teams learn from each other and learn about quality improvement methods from independent experts; a second team of leadership consultants to work with the project teams on team development and leadership skills; and a third team from RAND Europe and the Health Economics Research Group at Brunel University to undertake the external evaluation of the initiative as a whole (which included support for the self-evaluations of each project).

This paper describes the development of the protocol for the external evaluation.

### The invitation to tender for the external evaluation of the EwQI

The Health Foundation provided £4.3 million for the initiative. Following the call for proposals in September 2004, the Invitation to Tender for the external evaluation (ITT) was issued in February 2005. By this time six of the final eight EwQI projects had been commissioned.

The ITT outlined the scope, scale, and ambition of the EwQI, and the corresponding complexity of the proposed two-level evaluation. It stressed the need for interaction between the external evaluators and the project teams: the external evaluators were expected to work with the project teams on the development and implementation of their self-evaluation plans, and the project teams were required to participate in all aspects of the evaluation at both project and initiative level (Table 3). At both levels, the evaluations were expected to determine progress against the EwQI objectives, identifying and measuring outcomes, assessing the processes adopted, and exploring the thinking behind the projects in order to identify 'the factors associated with success'. But there was a difference in scope: the external evaluation was expected to address all three EwQI objectives, whereas the project self-evaluations were to focus mainly on the second.

**Table 3:** Objectives of EwQI evaluations

**Objectives of EwQI external evaluation (reflecting all EwQI objectives)**
1. To measure the extent to which patient care has improved.

2. To assess the level of increase in professional engagement in clinical quality improvement.

3. To measure the effectiveness of the initiative in leveraging external commitment to clinical leadership of quality improvement.

4. To evaluate the increase in competency and infrastructure for quality improvement in the professional bodies.

5. To assess the policy influence of the initiative.

**Objectives of project self-evaluations (primarily reflecting second main EwQI objective – as in Table 1)**

1. To assess the extent to which individual projects achieve measurable improvements in patient care.

2. To identify the factors facilitating/hindering change.

3. To evaluate improvement interventions.

4. To evaluate the proposed audit.

5. To participate in all aspects of the external evaluation.

The ITT listed six 'aims' for the external evaluation (Table 4 (with related tasks later identified by the evaluation team)). These aims confirmed The Health Foundation's intention that the external evaluators should work with the project teams to measure improvements in patient care through their self evaluations, rather than duplicating these measurements.

**Table 4:** Aims of the EwQI external evaluation (with related tasks identified by the external evaluation team)

**Aim one: to work with award holders on the development and implementation of their evaluation plans** **Tasks** - work with the project teams to support their self-evaluations, including data identification and validation. - assess the experiences of the users as 'active partners' in the projects, seeking to establish, for example, their role in defining outcome measures and their contribution to the design and implementation of improvement interventions and to governance arrangements. - consider how the counterfactual for each project can be addressed to assess how much change was attributable to the project, and how much to secular activity.
**Aim two: to synthesise the data and findings from project level evaluations** **Task** - synthesise the data and findings from project level evaluations using a modified form of logic modeling within an overall framework informed by realist evaluation and develop a logic model for the initiative as a whole.

**Aim three: to assess increases in clinical engagement in quality improvement** **Tasks** - gauge current clinical engagement through an examination of the documentary evidence, using the projects' original proposals and other evidence made available to us by the projects. - following this, conduct interviews with project team members and key informants in order to explore the state of affairs in the quality improvement context of each project before it has had a chance to influence that setting. - assess the change achieved during the life of the initiative by supporting each project in designing, implementing and analysing a survey of relevant clinicians. - in the final year of the initiative, conduct a web-based Delphi survey to identify how clinicians can best be engaged in quality improvement initiatives.

**Aim four: to measure the effectiveness of the award scheme (during its life) in leveraging external commitment to clinical leadership of quality improvement** **Tasks** The results of the project surveys and the Delphi will be used to support a workshop with representatives from each project on leveraging external commitment, identifying barriers, facilitators, processes, and outcomes.

**Aim five: to evaluate the increase in competency and infrastructure for quality improvement in the professional bodies involved in the EwQI** **Tasks** - conduct in-depth interviews with each relevant professional body focusing on the issues identified by Leatherman and Sutherland, *viz*: standard setting, development of quality measures, data collection and analysis, peer review and the design, based on evidence, of interventions to predictably improve patient care. - look at what the professional bodies involved in the EwQI have done. How effectively have they involved users? Have they promoted more effective use of audit and of audit data?

**Aim six: to assess the policy influence and cost consequences of the initiative** **Tasks** **1. Influence of the EwQI** - evaluate the projects' legacy plans - ask the project teams to identify the impact their work has had on the development and implementation of other quality initiatives, such as, for example, the development of a relevant NSF. **2. Cost consequences** - work with the projects to explore what data they can provide to estimate costs. - provide further advice on these requirements to the project teams - collect data throughout the EwQI on the 'central' costs of the initiative

The ITT also provided brief, one-paragraph summaries of the six projects already commissioned. These highlighted the variation between the projects in terms of the clinical problems they planned to address, and in approach and scope. There were also differences in timing, start dates ran from April 2005 to November of that year, and in duration, which ranged from three to four years. In addition, there was variation within each project – all the project teams planned to recruit large cohorts of participants from different sites across the NHS to implement their selected improvement interventions. Table 5 lists the eight EwQI projects, and more information is available on The Health Foundation's website [16].

**Table 5:** EwQI projects (initial plans)

**Lead organisation**	**Project**	**Study design**	**Scope**	**Duration**
Imperial College and Association of Coloproctologists of Great Britain and Ireland	To improve the quality of care for patients with cancer of the large bowel	Audit and feedback Time series analysis of repeat audits	Building on existing on-going national audit, aiming for 100% participation. 105 contributing hospitals	three years

Royal College of Physicians of London	To improve the care of patients admitted to hospital with exacerbations of chronic obstructive pulmonary disease	Audit and feedback A complex randomised controlled intervention with multi-professional paired peer review	Building on a previous one-off national audit of 94% of UK acute hospitals. Aiming to recruit 100 participating sites	three years

Royal College of Physicians of London	To assess and improve services for people with inflammatory bowel diseases	Audit and feedback Comparing three approaches, time-series but no control	Developing a national audit. Aiming to recruit 80% of all (approx 240) acute Trusts	four years

Royal College of Nursing	To improve the care of adult patients across the U.K. undergoing surgery by implementing national clinical guidelines on perioperative fasting	Audit and feedback Randomised study of three modes of disseminating an educational package (passive, interactive web-based, PDSA) Time series analysis	30 participating Trusts	three years

Royal College of Physicians of Edinburgh	A two-armed project to improve the management of community acquired pneumonia (CAP) and epilepsy	Double audit cycle with feedback, time series but no control	For CAP – half the Scottish Health Boards For Epilepsy – over one third of all Scottish practices and five clinics	three years

Royal College of Psychiatrists	To improve services for people who have self harmed	Time series analysis of repeat audits	34 selected teams	four years

Royal College of Psychiatrists	To improve prescribing practice for patients with serious mental illness	Time series analysis of repeat audits	40 participating Trust and two private healthcare organisations. Aiming to expand this number throughout the project	four years

Royal College of Midwives	To improve the quality of clinical care in the assessment, repair and the short and longer-term management of second degree perineal trauma	Audit and feedback Paired cluster randomised trial to establish effectiveness and persistence of training package	10 paired units	three years

But when the ITT was issued, there were no further details. This meant that if, as The Health Foundation intended, the evaluation was to start at the same time as the projects, the evaluation protocol had to be written with very limited knowledge of six projects, and none at all of the other two. On the other hand, it was also clear that subsequent deeper understanding of the projects (and of the EwQI itself) would inevitably influence our approach. To this extent, the evaluation protocol had to be developmental.

### The initial EwQI evaluation protocol

In our response to the ITT we drew on the relevant literature. This included UK government policy on quality improvement in the health service and the literature on which that was based, such as the Report of the Bristol Inquiry and related papers [17]. We also looked at the work of the US Agency for Healthcare Research and Quality and the US-based Institute of Healthcare Improvement, identifying key documents such as the Institute of Medicine's 'Crossing the Quality Chasm' [18]. Across disciplines, we looked at papers from a range of research fields, including research implementation [11], clinical audit and its use [19], clinical governance and user involvement [20], teamwork in healthcare [21] and organising for quality [22,23], the impact of research [24], and evaluation itself [13].

In the light of the above, we then reconsidered the immediate intellectual context cited by The Health Foundation, and identified the key themes in Leatherman and Sutherland's analysis that we thought were particularly relevant to the EwQI (Table 6).

**Table 6:** Key themes in Leatherman and Sutherland

• Clinicians work in the NHS as members of clinical teams, not as isolated individuals
• Work of these teams is, in turn, strongly influenced by the (local) organisational culture

• Importance of professional bodies in supporting quality initiatives in the NHS

• Huge difficulties in measuring cultural change, especially when there are multiple cultures and sub-cultures, hence the inadequacy of the current evidence base and the need for rigorous evaluation

• Importance of user involvement in quality improvement

• Importance of sustaining quality improvements and, hence, of participative rather than top-down approaches.

The need to explore change at many levels and in many contexts, and to explore the values, knowledge, and roles of all those involved shaped our methodological approach. The brief for the evaluation was not only to establish 'what worked' but also to understand why it worked (or failed to work), *i.e. *what worked, in what contexts, and for whom. We concluded that the external evaluation had to be methodologically pluralistic. Using an experimental design for the external evaluation was not our preferred option for the reasons set out above, and in any case, was not available because the EwQI had already been designed, and most of the projects had been commissioned. We therefore proposed an approach based on logic modeling within a framework informed by realist evaluation, in order to capture and use information about why the projects were working (or not) [13,14].

Realist evaluation aims to establish clear and measurable relationships between a project and its outcome. It assumes that there is an underlying theory of change behind the project explaining how it brought about the measured change and is sensitive to the context in which the project is delivered, identifying a series of Context-Mechanism-Outcomes (CMOs) for each intervention. One difficulty with this approach is that any intervention can have a large number of CMOs [25]. We planned to use the professional, tacit, and formal knowledge of the EwQI project teams to narrow this number, working with them to develop illustrative logic models for each project and to identify those aspects of their projects that they regarded as important in achieving improvement in clinical care. Table 7 shows a hypothetical logic model for an EwQI project.

**Table 7:** A hypothetical framework for a logic model in EwQI

Situation: lack of clinical engagement is compromising clinical quality				
Context	Mechanisms		Outcomes	
IF →	THEN IF →	THEN IF →	THEN IF →	THEN
Department or practice is involved with EwQI →	Clinicians will engage with quality more fully than before →	Teams' behaviour will be more patient focused →	Health outcomes will improve →	Clinicians will become committed to engaging with quality

Within this framework, we took the six aims in the ITT and identified a series of tasks under each aim (Table 4), with an accompanying GANTT chart that showed what we intended to concentrate on during each year of the evaluation. There was some overlap between the six aims, and this was reflected in links between the component tasks. In July 2005 we reached agreement with The Health Foundation on our initial protocol. This included agreement that the evaluation protocol was 'emergent', *i.e. *still under development, and would be finalised at the end of the first year of the EwQI.

### The first year

In the first (developmental) year of the evaluation we concentrated our activities on aims one, two, and three, with some input to aim six (interpreted to include a cost-consequences assessment of the initiative). No formal input was planned to aims four and five during this early period. This section describes what we did, outlining some of the problems encountered and the solutions adopted during this first year.

### Aim one: to work with award holders on the development and implementation of their evaluation plans

#### Problem one: understanding the EwQI

The Health Foundation intended the EwQI to be an emergent initiative, in which the improvement interventions implemented by the project teams were clarified through an iterative process of action and reflection. This developmental approach was innovative, and it came as a surprise to the project teams. Initially they were unclear about how much time it would involve, and those committed to what they saw as relatively straightforward research or audit projects were unconvinced about its value. There were also confusions about the roles of those providing support and evaluation. All this provided a difficult context for our initial meetings with the project teams. A major task was to gain their confidence and together explore how we could all best exploit the opportunities for reflection and development that the EwQI provided.

##### Solution

Through numerous interactions during the first year (some formal, some informal), we sought a shared understanding with the project teams, with The Health Foundation, and with the support team about the EWQ1, the project teams' roles, and the contexts within which each project was working. We also explored the skills and experience available to the teams, the intended outcomes of each project, and the mechanisms (improvement interventions) each project team had chosen to achieve those outcomes. We used logic models (initially drafted by the external evaluation team) to explore the thinking behind each project. We discussed the relation between the project self-evaluations and the external evaluation. The aim was to encourage a reflexive approach among all those involved, including ourselves, through which evaluation could contribute to learning and to changes in practice.

#### Problem two: understanding quality improvement

Quality improvement is not a pill administered as a standard dose in a controlled setting to passive recipients while a control group receives a placebo [26]. It relies on complex interventions (training programmes, audit and feedback, guidelines, etc.) undertaken in local contexts and aimed at active participants who bring with them a whole baggage of values, attitudes, and preconceptions about present practice and the possibilities of improvement. The need to build on small local changes is increasingly recognised [27]. This involves implementing improvement interventions bit by bit, building on and learning from previous gains – repeat audits, plan-do-study-act cycles, interactive training programmes, etc. Our initial meetings with the EwQI project teams confirmed that their backgrounds and their interpretation of the EwQI varied widely. Some project teams saw themselves as researchers, others as clinicians developing clinical audit, others as members of established departments in professional bodies dedicated to improving the quality of care. Project design reflected these differing views, ranging from research studies to audit to the development of training programmes, or various combinations of the three. There was, in other words, no common view *ab initio *about the best means of engaging clinicians in quality.

##### Solution

To promote a clearer, shared understanding of quality improvement, the support team organised a series of initiative-wide meetings that covered topics such as quality measurement, team development, change management, audit practice, user involvement, and communication plans. The requirements of both levels of evaluation were also considered.

#### Problem three: nature of the evaluation

We also found confusion among the project teams about the nature and timing of the two-level evaluation, and about their own role in it. It emerged that the teams had been largely unaware of the evaluation when they signed up, and had not considered it (as a possible constraint) when project plans were being developed. And even when they were made aware of The Health Foundation's requirements, not all the teams appreciated that evaluation was intended to run alongside the initiative as it developed. Many of them saw evaluation as something to be done at the end of the project, something that could wait until later. There were uncertainties about the nature of external evaluation (were we there to judge, or to help?), and a limited appreciation of its broad scope. Evaluation methodologies, such as theory-based evaluation and logic models, were new to many of the teams. Only two project teams mentioned any form of economic assessment in their original proposals, and in general, the teams' views on evaluation tended to be shaped by an emphasis on clinical outcomes and a tendency to see the EwQI in terms of either research or clinical audit.

##### Solution

In response, we worked with The Health Foundation to produce detailed guidance about what was required in the project self-evaluations, including a set of nine questions which we asked the teams to address in their first self evaluation returns (Table 8). This had the dual benefit of enabling us to clarify these requirements with The Health Foundation, and of providing us with a tool through which to discuss them with the project teams. The guidance also explained the interactions between the project self-evaluations and the external evaluation. We held a second round of meetings with the teams to discuss the guidance, and also provided detailed briefing on some of the more technical aspects where the teams told us they needed help, such as cost consequences analysis (aim 6, Table 4).

**Table 8:** Key questions to be answered in the EwQI self evaluations

**Q 1. Background**	• Why was this project needed? • Why did you think that your approach would be effective? • Did you consider other approaches? If so why were these rejected? • What was the project team's understanding of the self-evaluation and its purpose? Did this change during the project?
**Q 2. Process – what improvement intervention was introduced to whom and how?**	• What did the project team do? • Who did they involve? • How were these activities evaluated?

**Q 3. Outputs**	• What did these activities produce? • How were these outputs evaluated?

**Q 4. Who did what**	• Who was involved in designing, implementing and evaluating the project? What was their contribution?

**Q 5. Outcomes – did the project work?**	What did these activities achieve in terms of: • Measurable improvements in patient care • Increase in the levels of professional engagement in QI • Increase in the capacity and infrastructure for QI in the professional bodies involved in the project • Increase in the knowledge base • Sustainable arrangements for improving quality of care in this field of medicine? How were these changes measured?

**Q 6. What difference did the project make?**	• The EwQI is only one of a number of initiatives currently addressing quality improvement in the UK health system generally and in particular specialties, how much difference was really made by the project itself in the context of all this other work?

**Q 7. What are the cost consequences of the project?**	• Without attempting to provide a monetary value to the outcomes of the project, how much did the project cost in real terms and with what benefits? Could this have been achieved more easily in other ways?

**Q 8. Why did the project work?**	• Factors that helped/hindered • How were clinicians and patient groups engaged and with what consequences? • What were the key ways of bringing about change (*e.g*. repeat audit, training, information provision) and how well did these work? • Could the project be seen to have worked for some people but not for others?

**Q 9. What arrangements are in place to ensure the sustainability of the project's work?**	• How might the result of the project 'fit' with wider changes (*e.g*. in the professions, funding, training, organisational context)?

#### Problem four: what would have happened anyway – did the projects cause the outcomes they identified?

Each EwQI project team planned to involve large numbers of participating units (20 in one project, over 190 in another), each of which provided a different context for quality improvement. All the project teams planned to support clinical audits in participating units, with central analysis of audit data and feedback to participants, and time series analysis to establish clinical outcomes. To address the question of whether the project had actually caused the identified changes, three research-oriented project teams also intended to introduce a form of randomised control. For example, one team planned to use a randomised cluster design allocating participating units to separate arms of the study, one of which would receive the improvement intervention (a training programme) early, the other at a later stage. The remaining five audit-orientated projects planned to use time-series assessments, but with no controls. One of the latter teams was developing an established audit already aiming for 100% inclusion which meant that, although this team already had several existing rounds of data and could identify trends in improvement during the lifetime of the audit, they had no means of unequivocably attributing that improvement to the audit. Therefore, some of the project teams were better placed than others to establish whether the outcomes they identified could be reliably attributed to their project.

## Discussion

Coming from different backgrounds and working in different contexts, the project teams interpreted the EwQI brief in various ways, each taking the approach they thought would best achieve their identified objectives. This diversity was integral to the EwQI, and one of the things it was set up to explore. Reflecting the debate described at the start of this paper, there was no general view among the project teams that one methodological approach was better than other, possibly multiple, approaches. As external evaluators, our task was to unpack each team's assumptions and assess to what extent their approach was fit for purpose, comparing and contrasting those approaches across the initiative. As advisors on the project self-evaluations, we also sought to enhance what the teams were doing, stressing the importance of understanding and describing local and external confounders, even if they could not 'control' for them.

### Problem five: ethics review

Formal ethics review is a requirement for all research involving patients, and is usually handled by award holders as a routine part of getting a project up and running. Quality improvement involves mixed approaches, and often includes research. In the EwQI we found that the project teams had differing views about the need to get ethics approval: the research-orientated teams were certain that it was necessary; the audit-orientated teams were equally convinced it was not needed. A number of teams were also concerned that ethics review was causing delay.

#### Solution

With the support team, we approached the UK Central Office for Research Ethics Committees (COREC, now the National Research Ethics Service) to clarify matters. Ethics review procedures are designed to protect patients involved in research from undue risk. We found an ongoing debate about the scope of these procedures in the UK and in the US [28,29]. Should they apply to all research projects in the same way? Should they apply to audit, service evaluation, and quality improvement programmes [30]? Like research projects, quality improvement programmes are not without risk, but then much medical practice also involves risk [31,32]. What is important is the level of risk experienced by patients involved in a project [33]. All the EwQI projects were undertaking clinical audits, which are exempt from ethics approval [28]. However, audit projects that contain elements of research require approval [34]. The key distinction is still level of risk. In some instances approval was required, in others not: there was no one case fits all. For the external evaluation COREC determined that approval was not needed.

### Aim two: to synthesise the data and findings from the project level evaluations

#### Problem one: data collection

All the project teams planned to measure clinical outcomes. Half the project teams also planned surveys or interviews of clinicians (to explore their attitudes to audit and quality improvement), three out of eight teams planned surveys or interviews of users and caregivers (to explore their perceptions of care and its outcomes), and two projects intended to collect costing data. We agreed with The Health Foundation that it would be counterproductive to duplicate these activities. Our main focus was therefore on data collection through the projects, and on ensuring that we had access to the results of the project teams' analyses. But we also needed to establish any significant gaps in the data that the teams planned to collect, such as data on costs, and explore how these gaps could be addressed.

##### Solution

The self-evaluation guidance identified the data requirements of both levels of evaluation. Using this we discussed these requirements with the project teams, and explored how identified gaps could be remedied.

#### Problem two: synthesising the data

To develop an overview, we planned to synthesise the data from the project self-evaluations using a generic logic model as an explanatory framework. The aim was to illustrate how – at various levels within the health system and among all the participants involved – initiatives such as the EwQI influence prior determinants such as beliefs, values, and patterns of behaviour to produce changes in clinical and non-clinical outputs. In line with The Health Foundation's developmental approach, this was planned to be an iterative and reflexive process, developed collaboratively with The Health Foundation and the project teams. But when we met the project teams and discussed their plans in detail, we found much more variation between and within the projects than we had expected at the outset. It looked increasingly unlikely that we would find one organising framework within which to synthesise all the findings from the projects, *i.e. *one overarching logic model as we had originally planned.

##### Solution

We concluded that drawing together the findings from the projects could not be a simple aggregation of evidence. The EwQI is multi-project, multi-site, and multi-method, and raises evaluation problems akin to the challenges of programme evaluation. We are using the self-evaluations of the projects to generate theories. We will consider and weight the evidence provided by the projects to support or weaken these theories. We will then compare and contrast common theories across the initiative to generate more fine grained and conceptually rich interpretations of what works in what circumstances.

### Aim three: to measure increases in professional engagement in clinical quality improvement

#### Problem

Half the project teams planned surveys or interviews of clinicians to explore their attitudes to audit and quality improvement, and during the first year we were able to encourage three others to undertake some form of survey. These mainly concentrated on clinicians' confidence about the management of a particular clinical condition. But the information requirements of the external evaluation were broader, concerning clinicians' attitudes to and engagement with quality improvement in general.

##### Solution

We asked the teams to extend their surveys so that they met the information requirements of both levels of evaluation. We also planned from the outset to undertake our own web-based Delphi survey [35] of participating clinicians towards the end of the initiative in order to identify: how clinicians can best be engaged in quality improvement initiatives; what impact this is thought to have on clinical outcomes; and how this work best interfaces with the engagement of patients, other professionals and health services managers to leverage external commitment to clinical leadership of quality improvement.

### Aim six: to assess the policy influence and cost consequences of the initiative

#### Problem

In the original ITT for the EwQI, the teams had been asked to consider the sustainability of their projects, including their influence on policy. We agreed with The Health Foundation that this aim should include a cost consequences assessment of the initiative, and that the project teams should be asked to undertake a simple cost consequence analysis, quantifying the resources used to promote quality improvement and the main quantitative outcomes. Initially only two teams planned to collect any cost data.

##### Solution

Using the self evaluation guidance, we discussed all these requirements with the project teams, exploring what data they would be able to collect and appropriate methods of analysis.

### Finalising the protocol

We have described how we worked with the project teams during the first year to explore the objectives of the EwQI, its two levels of evaluation, and their own projects, in order to develop a common understanding. We have also described how the variation between the projects – in approach, scope, and context (including the support provided by the parent organisation) – was much greater than we expected at the outset. Was an opportunity missed to impose a common approach on the projects? We think not: their diversity was illustrative of various approaches to quality improvement found more generally, and reflected in the debate on methodology and epistemology mentioned at the start of this paper. And it was this diversity that the EwQI had been set up to explore: *i.e. *what was the starting point of the Royal Colleges and professional organisations involved, and how effectively were they able to support their members in engaging in quality improvement? But, as discussed, this variation challenges our attempts to synthesise findings from the projects.

The project self evaluations got off to a difficult start, many of the project teams were initially unconvinced about the need for evaluation. A clearer statement of the requirements for this when the projects were being formulated would have helped. We also found that the terms lacked experience of, and/or were unconvinced by, some of the methodological tools we encouraged them to use. Most teams had not previously undertaken a cost consequence analysis, although all could see its relevance and were eager to learn more. The teams made little use of their logic models, seeing them as a high-level narrative summary, rather than as a systematic and detailed way of reflecting on their projects. During the first year, we developed a supportive relationship with project teams through one-to-one meetings and by providing extra help where necessary, *e.g. *with ethics review. In the light of the teams' lack of interest in logic models, we are using their self evaluation returns to highlight the key theories underpinning each project and explore these with them.

We had early agreement with The Health Foundation that data collection would be undertaken through the project self evaluations. This was helpful – it focused our minds on what we needed from the self-evaluations, and on the necessary interactions between both levels of evaluation. During the first year, we developed the self-evaluation guidance and, as described above, this has been a useful tool.

There were two more general difficulties. One was keeping a balance. As external evaluators of the whole initiative, we needed objectivity. But we were also required to help the project teams develop and implement their self-evaluations, and to do this we needed empathy and close engagement with the teams. These approaches are incompatible. The Health Foundation's steer was towards the second, but we needed to balance one against the other. The other constraint (though it was also an opportunity) was the developmental approach adopted by The Health Foundation: this meant that we were assessing a deliberately changing picture, a process of reflection and change.

We appreciated from the outset that we would be evaluating not one but a series of complex social changes, and that our protocol would need to reflect this. Our overall approach was methodologically pluralistic and, although it was influenced by what we learned from the projects during the first year, largely remained unchanged. Some of the tools we had planned to use, such as the logic models, proved less useful than anticipated; others, such as the self evaluation guidance, were developed in response to the needs of the projects.

## Conclusion

The approach The Health Foundation took in the EwQI was innovative: learning and development were integral to the initiative, and evaluation was built in from the outset. This approach was unfamiliar to project teams more used to working on research and/or audit projects. The concepts and practice of quality improvement were also unfamiliar to many in the teams. Due in large part to the efforts of the support team, the first year of the EwQI saw considerable gains in understanding about what the initiative was trying to achieve and about quality improvement in general. In a number of projects these new insights were associated with subsequent changes in the design and/or implementation of the project itself. Our own understanding of the EwQI and the context in which it had been established developed alongside that of the project teams.

In such a fluid situation, a rigid evaluation protocol implemented unchanged from the start of the EwQI would have been inappropriate. The emergent approach we developed with The Health Foundation's agreement proved not only necessary but also, we would argue, essential if, through development and evaluation, changes in clinicians' attitudes to clinical engagement in quality improvement are to be identified and encouraged.

This paper has described the protocol for the external evaluation of the EwQI, and the way in which that protocol was shaped by interaction with the project teams during the first year. Our experience has been that this developmental approach enhanced the capacities of all involved to reflect on the EwQI and seek to use evidence better in engaging clinicians and delivering improvements for patients and for the health care system. It should lead to a more textured, informed, and modulated final evaluation.

## Competing interests

The authors declare that they have no competing interests.

## Authors' contributions

TL, SH and BS devised the original protocol for this evaluation, and all the authors played a considerable role in developing the finalised protocol. BS led on the production of this article, and all the authors have read and agreed the final manuscript.
